# Mangrove dieback during fluctuating sea levels

**DOI:** 10.1038/s41598-017-01927-6

**Published:** 2017-05-10

**Authors:** Catherine E. Lovelock, Ilka C. Feller, Ruth Reef, Sharyn Hickey, Marilyn C. Ball

**Affiliations:** 1 School of Biological Sciences, The University of Queensland, St Lucia, Queensland, 4072 Australia; 2 Smithsonian Environmental Research Center, Edgewater, Maryland 21037 USA; 3 School of Earth, Atmosphere and Environment, Monash University, Clayton, Victoria 3800 Australia; 4 School of Earth and Environment Sciences, Oceans Institute, University of Western Australia, Crawley, Western Australia 6009 Australia; 5 Research School of Biology, The Australian National University, Acton, Australian Capital Territory 2601 Australia

## Abstract

Recent evidence indicates that climate change and intensification of the El Niño Southern Oscillation (ENSO) has increased variation in sea level. Although widespread impacts on intertidal ecosystems are anticipated to arise from the sea level seesaw associated with climate change, none have yet been demonstrated. Intertidal ecosystems, including mangrove forests are among those ecosystems that are highly vulnerable to sea level rise, but they may also be vulnerable to sea level variability and extreme low sea level events. During 16 years of monitoring of a mangrove forest in Mangrove Bay in north Western Australia, we documented two forest dieback events, the most recent one being coincident with the large-scale dieback of mangroves in the Gulf of Carpentaria in northern Australia. Diebacks in Mangrove Bay were coincident with periods of very low sea level, which were associated with increased soil salinization of 20–30% above pre-event levels, leading to canopy loss, reduced Normalized Difference Vegetation Index (NDVI) and reduced recruitment. Our study indicates that an intensification of ENSO will have negative effects on some mangrove forests in parts of the Indo-Pacific that will exacerbate other pressures.

## Introduction

Mangrove forests are comprised of halophytic woody species whose distribution is strongly influenced by tidal and freshwater inputs that maintain gradients in soil moisture, salinity, anoxia and nutrient availability^1^. Inundation with saline water introduces salts into the soils, and these salts become concentrated in the soil porewater when water is lost from the soil by evapotranspiration. Where there is limited land and/or atmospheric-derived fresh water sources, as occurs over much of the seasonally dry tropics, evapotranspiration can lead to very high concentrations of salt in soil porewater in the intertidal zone which can exceed the salinity tolerance of mangrove trees. For example, long-term studies indicate that variation in rainfall and sea level can have enormous influences on the landward extent of mangroves, which expands during periods of high sea level and high rainfall and contracts seaward when sea level is low and rainfall is diminished^2^. In the tropical Pacific Ocean, ENSO can result in extreme variation in sea levels. During El Niño, weak equatorial trade winds cause the thermocline to shoal in the tropical western Pacific and the presence of cool water results in sea levels that can be lower by 20–30 cm, while conversely sea levels are higher in the east^3, 4^. During the La Niña phase the patterns are reversed. These sea level seesaws are intensifying with climate change^5–7^ and are therefore likely to increase fluctuations in tidal inundation and salinity of mangrove soils, giving rise to conditions unfavorable for tree growth. Using unique long-term monitoring of a site at Mangrove Bay in north Western Australia, we provide evidence that extremely low sea levels during recent intense El Niño events^4–7^ have led to mangrove dieback.

**Figure 1:**
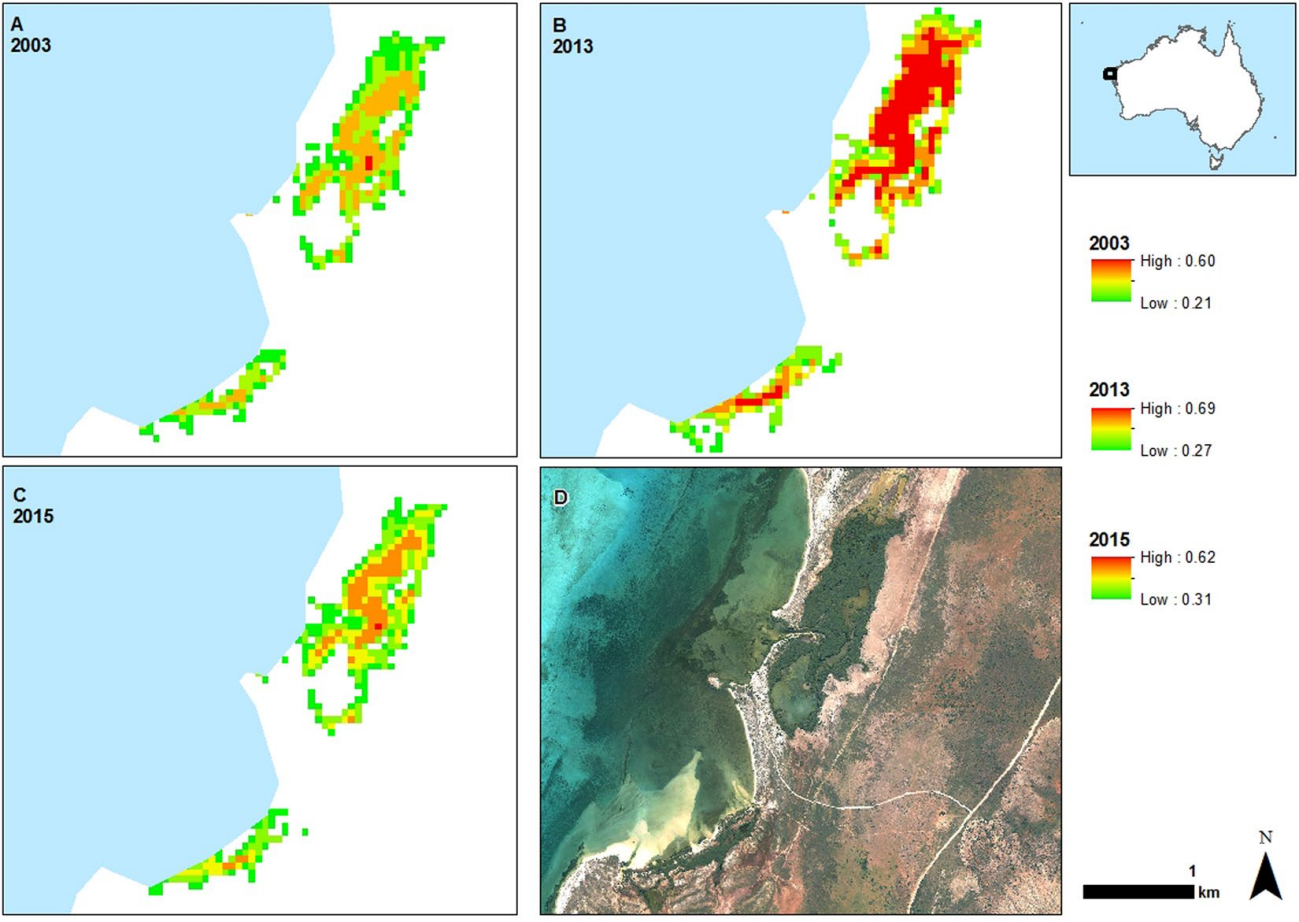
Normalized difference vegetation index (NDVI) of Mangrove Bay mangrove forest showing the thinning canopy during the dieback in 2003 associated with low sea level and high salinity of soil porewater (**A**), after recovery of the canopy in 2013 (**B**), and after the most recent downward swing of the sea level seesaw in 2015 (**C**). An aerial image of the site (**D**) shows the mangrove fringing small creeks and lagoons adjacent to the Ningaloo reef flat and the grassland dominated terrestrial environment. The location of Mangrove Bay on the Australian coast is shown in the inset. NDVI was obtained from Landsat scenes (Table S1), the aerial image from 2012 was obtained from © 1995–2016 Esri (Service Layer Credits: Source Esri, Digital Globe, GeoEye, Earthstar Geographics, CNES/Airbus DS, USDA, USGS, AEX, Getmapping, Aerogrid, IGN, IGP, swisstopp, and the GIS User Community). Geographic boundaries represent Australian Statistical Geography Standard (ASGS) provided by the Australian Bureau of Statistics (ABS, 2011, data freely available at http://www.abs.gov.au/AUSSTATS/abs@.nsf/Lookup/1259.0.30.001 Main+Features1July%202011?OpenDocument). Map generated in ArcMap v10.3.1.

Our site is a mangrove-lined bay adjacent to the Ningaloo Reef in north Western Australia which is dominated by the widespread mangrove tree species *Avicennia marina* (Fig. 1). The region is arid, with low rainfall and high evaporative demand leading to hypersalinity of soil porewater in high intertidal mangrove habitats and adjacent cyanobacterial mats, salt marshes and salt flats. Despite its small size (40 ha) the mangrove is highly significant due to its importance to biodiversity in the region, including providing habitat for fish, birds and terrestrial vertebrates, and for its importance for tourism^8–10^. We have monitored the site since 2000 during which two episodes of canopy dieback were observed, one in 2002–2003 and the other in 2015–2016 (Fig. 1 and Supplementary data Fig. 1). The second of these dieback events, which was more extensive than the event in 2002–2003 was coincident with the large-scale death of mangroves along the southern coast of the Gulf of Carpentaria in northern Australia^11^ and may provide some insight into the underlying causes of this larger scale event.

**Figure 2:**
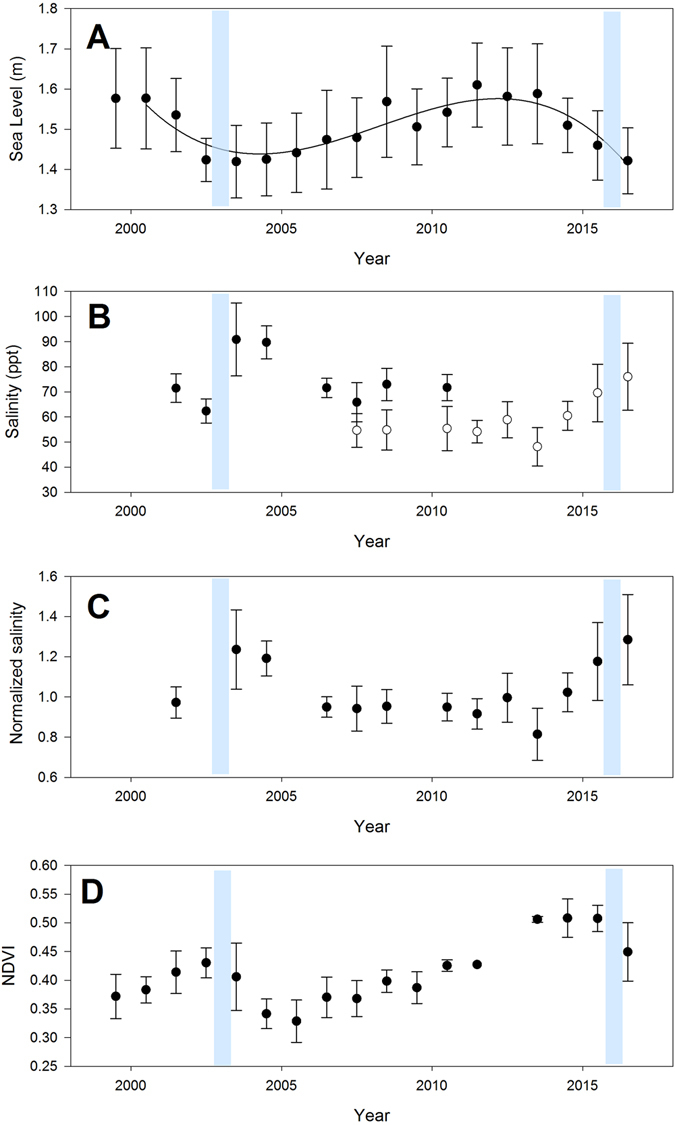
Inter-annual variation in mean sea level (**A**), mean soil porewater salinity of two monitoring campaigns (black and white symbols) (**B**), normalized salinity (**C**) and mean annual Normalized Difference Vegetation Index (NDVI) **(D)** from 1999–2016 at Mangrove Bay on the Ningaloo coast, Western Australia. Error bars are standard deviations. Salinity was normalized (expressed in relative units) to account for differences in locations of the two monitoring strategies over the study period. A value of 1 is equivalent to the initial value while 1.25 indicates a value 25% higher than the initial soil salinity. Elevated soil porewater salinity was evident in 2003 and 2004 as well as 2015 and 2016, both periods which were associated with wide spread canopy dieback (indicated by the blue bars). Source data are available in Supplementary data Table 2.

**Figure 3:**
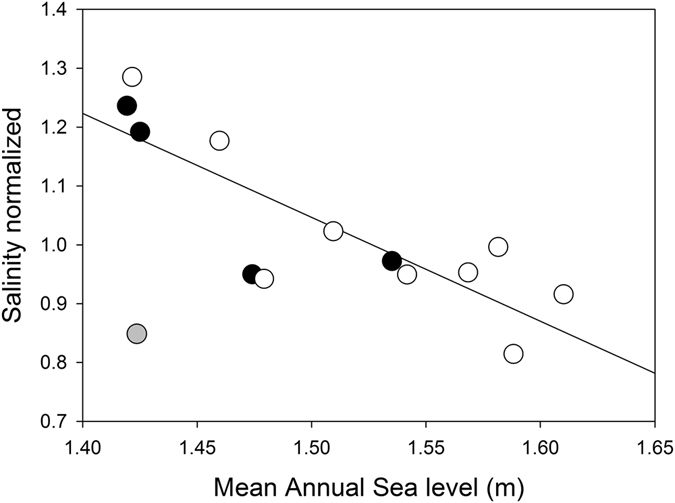
The relationship between normalized porewater salinity and the annual mean sea level over the study period. The regression, of the form y = 3.70 + −1.77 * x, R^2^ = 0.69, is significant either with or without the data for 2002 data included (indicated in grey) which was a year with intense rainfall in the month prior to sampling. Filled symbols are from 2002–2011 and open symbols from 2007–2016.

Mean annual sea level obtained from the closest tide gauge (see Methods) varied 20 cm over the study period (Fig. 2). It was high in 2000 and 2001, dropping 20 cm to a low between 2002 and 2004 when the first dieback of the forest was observed. It peaked in 2011–2012 before declining in 2015–2016 when the second dieback of the forest was observed. Soil porewater salinity also varied over the 16 years of monitoring. In 2003–2004 porewater salinity was approximately 25% higher than mean levels, and in 2015–2016 it was elevated by 30%. The variation in mean sea level over the 16 years of monitoring was negatively correlated with normalized soil porewater salinity (Fig. 3), such that years of high salinity were associated with low mean sea level, indicative of lower levels of tidal inundation. The year 2002 was an exception, when low mean annual sea level was not associated with high soil salinity. This result may have reflected the high level of rainfall in May and June (179 mm of total 200 mm for the year) prior to the monitoring measurements. However, over the 16 year record, variation in annual rainfall or rainfall observed in winter or summer periods, had no significant influence on soil porewater salinity (Extended data Fig. 2).

**Figure 4:**
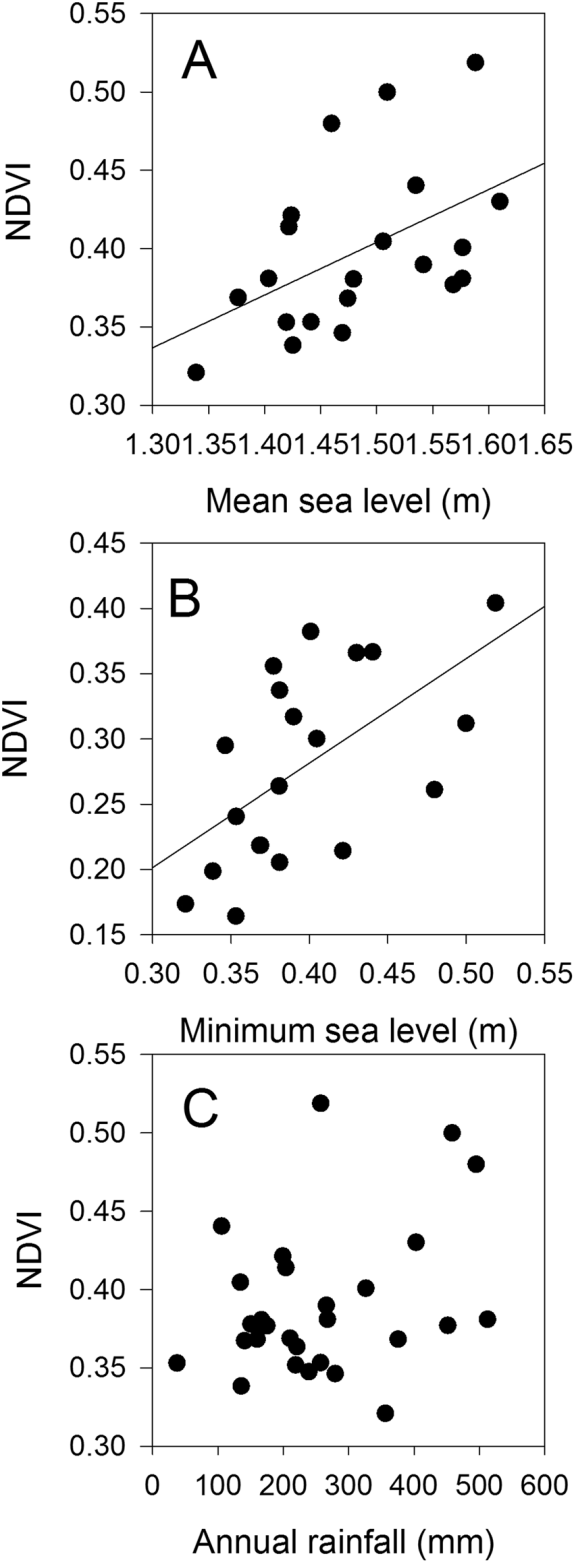
The annual mean Normalized Difference Vegetation Index (NDVI) of Mangrove Bay mangrove forest as a function of annual mean sea level (**A**), annual monthly minimum sea level (**B**) and annual rainfall (**C**). The regression line in panel A is y = −0.10 + 0.336 * x, R^2^ = 0.24 (P = 0.0246) and in panel B is y = 0.28 + 0.42 * x, R^2^ = 0.34 (P = 0.0074). The relationship between annual NDVI and rainfall was not significant (P > 0.05).

**Figure 5:**
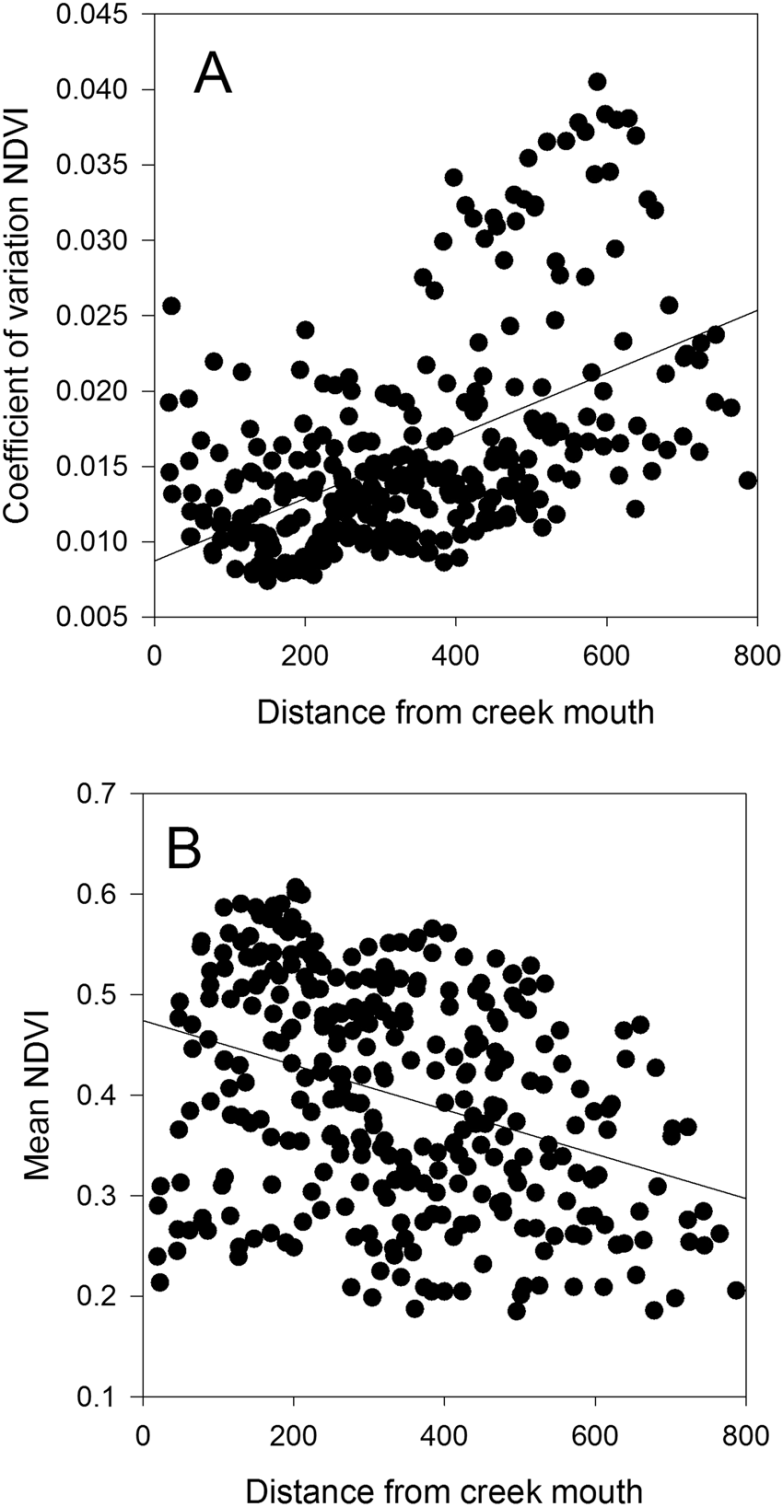
The coefficient of variation in the Normalized Difference Vegetation Index (NDVI) of pixels in the Mangrove Bay mangrove forest (**A**) and the mean NDVI (**B**) as a function of distance from the creek mouth. The regression line in panel A is y = 0.0087 + 0.000021 * x, R^2^ = 0.27 (P < 0.0001) and in panel B is y = 0.47 + −0.000022 * x, R^2^ = 0.12 (P < 0.0001).

Variation in the Normalized Difference Vegetation Index (NDVI, Fig. 1) over the study period also indicated strong reductions in NDVI in 2015–2016 and a smaller reduction in 2002–2003, although NDVI was low throughout the early 2000’s at the site, potentially reflecting the extended low sea levels associated with successive El Niño events in the 1990’s^12^ (Extended data Fig. 2). Over the entire available record of NDVI at the site (1987–2016), mean annual NDVI was significantly correlated with mean annual sea level and annual minimum sea level (Fig. 4), providing additional evidence that the forest was sensitive to annual variation in tidal inundation. However, there was no significant relationship between mean annual NDVI and normalized salinity values, due to the much higher NDVI prior to the 2015–2016 dieback compared to the 2002–2003 dieback event (Fig. 2). Analysis of mean NDVI including mean annual rainfall as an explanatory variable did not contribute significantly to explaining variation in NDVI over the 1987–2016 period (Extended data Table 3). In other locations in Australia, rainfall and sea level tend to co-vary with the El Niño-Southern Oscillation^12, 13^, but in the Pilbara region of Western Australia annual rainfall is not strongly reduced during El Niño events^14^, however sea level was lower^15^. There was significant spatial variation in NDVI over the 1987–2016 time series, with the coefficient of variation in NDVI for individual pixels being highest in locations most distant from the mouth of creek where the tide enters the mangrove forest (Fig. 5), suggesting trees furthest from the incoming tide and less likely to be inundated during low tide events, were the most likely to experience dieback.

**Figure 6:**
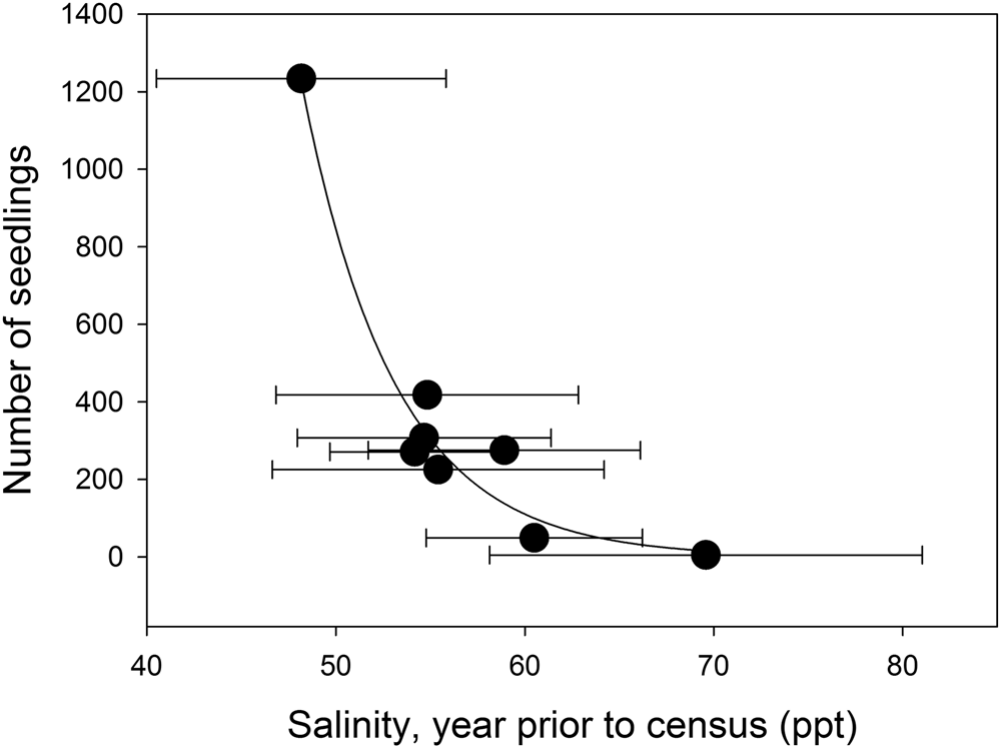
The relationship between the total number of seedlings observed in the monitoring plots and the mean soil porewater salinity (±standard deviation) for the year prior to the seedling survey (when propagules were developing). Seedlings were surveyed in 12, 5 × 5 m plots (total area of 300 m^2^). Curve is an exponential decay of the form y = 22,149,609 (±22,954,957) * exp (−0.2035 (±0.0208) * x), R^2^ = 0.96, where standard error of the parameter estimates is in parentheses.

Seedling recruitment was low during years with high soil porewater salinity (Fig. 6), indicating reduced reproduction (or dispersal) during periods of high salinity and low sea level. There was no relationship observed between annual rainfall and numbers of seedlings. Increases in the the NDVI was observed over 10 years between 2006 and 2016, coincident with increasing mean and minimum sea level (Figs 2 and 4). However, all plots, including those that were designated “live” in 2006 had suffered canopy losses of over 80% in 2016. Our monitoring of soil porewater salinity in 2016, within a broader survey of “live” and adjacent “dead” plots over the forest, indicated tree canopy loss was associated with higher mean soil porewater salinity of 68.5 ppt (±standard deviation 3.0) compared with 57.8 ppt (±SD 2.6) in “live” plots.

The intertidal position of mangrove forests gives rise to a high exposure to pressures arising from changes in both the ocean and adjacent terrestrial environments. While sea level rise due to a warming climate poses a significant threat to many mangrove forests^16, 17^, our data demonstrate that fluctuations in sea level, and particularly periods of low sea level, can also pose threats to mangrove forests in arid regions where inputs of fresh water is limited. As soil salinities rise, trees may lose their capacities for water uptake and salt exclusion and exhibit signs of stress consistent with those induced during drought^18–21^, including progressive loss of leaves, death of branches and eventually death of entire trees when salinities exceed tolerance limits^22, 23^. In many respects, the dieback of mangroves observed in response to increase in soil salinity is similar to other forest dieback events induced by severe drought^24^ in which suggested causes of tree death are highly controversial but consistent with hydraulic failure leading to catastrophic tissue desiccation^25^.

For mangrove forests, drought may increase the negative impacts of sea level fluctuations. Low sea levels may not cause mortality of mangroves if soil porewater salinity is ameliorated through freshwater inputs from rainfall or river flows, as may have been the case in 2002 in our data set. Because both low sea level and low rainfall co-occur during El Niño years in the Indo-Pacific region^26, 27^, intensification of ENSO in the coming decades with climate change may be particularly unfavorable for productivity of mangrove forest ecosystems. The recent extensive dieback of the mangrove forests in the Gulf of Carpentaria in northern Australia was associated with prolonged drought and high temperatures^11^, but it is also associated with the same 2015–2016 low sea level event reported here. There is evidence that mangroves can recover their canopy rapidly, over 10 years (Fig. 2) and expand in periods of high sea level and high rainfall^28^, although this may be limited to species that can recruit and grow rapidly^28^. Continuing intensification of ENSO with climate change and associated seesaw swings in sea levels combined with other stressors may therefore degrade mangrove forests, if the time between extreme events is not sufficient for forest recovery. Mortality of mangrove forests during intense episodic low sea level events may reduce the extent of mangrove ecosystems and lead to enhanced vulnerability to sea level rise as well as the loss of ecosystem functions and services, which include carbon sequestration, nutrient cycling, coastal protection and the provision of habitat for biodiversity.

## Methods

Our study site is within Mangrove Bay in the Cape Range National Park, Western Australia (lat. 21.97° S, long. 113.94° E). The mangrove forest is dominated by *Avicennia marina*, with *Rhizophora stylosa*, *Ceriops australis* and other tree species occurring at low abundance. The site has semidiurnal tides with a tidal range of 2 m. The density of trees in the tallest parts of the forest are 4400 stems ha^−1^ with a mean diameter of 7.9 cm^29^. In 2000, we began to monitor soil porewater salinity of 21 scrub trees (<2 m tall) in the southern side of Mangrove Bay, within a fertilization experiment. We measured the growth of these trees and the salinity of the soil porewater annually during the winter months (June – August) between 2001 and 2010, with the exception of 2005 and 2009. In October 2003, a dieback event was observed (Supplementary data Fig. 1) in which approximately 25% of the stand was affected (Fig. 1). Fertilization had no influence on soil porewater salinity or on observed tree dieback. In 2006, to more fully explore the dieback event of 2003–2004 and monitor recovery, we established 12, 5 × 5 m plots, with 3 pairs of “live” and “dead” plots in both the north and south of the Bay. Seedling recruitment and soil porewater salinity were monitored annually between 2006 and 2016, with the exception of 2009. In each 5 × 5 m plot the number and height of seedlings, saplings and mature trees was recorded each year. In July 2016, a new dieback event was observed (Supplementary data Fig. 1) in which approximately 30% of the trees died. In 2016, additional to our monitoring plots, we surveyed soil porewater salinity in 13 additional paired plots in the north of Mangrove Bay that were either live (>75% of canopy intact) or dead (<15% canopy remaining).

Soil porewater salinity was extracted from 30 cm in the soil using a suction device^30^ and measured with a hand-held refractometer. We calculated the mean soil porewater salinity for each year for both the fertilization experiment trees (*N* = 21) and the recruitment monitoring plots (*N* = 12). In order to obtain a single continuous record of salinity from both sites, we normalized salinity relative to the mean salinity for all years for those sites. Normalized soil porewater salinity varied from 0.81 to 1.28, where 0.81 indicates salinity is 19% lower than the mean value and 1.28 is 28% higher than mean value.

We obtained monthly sea level data monitored at the nearby coastal town of Exmouth (Bureau of Meteorology station ID 62435; Permanent Service for Mean Sea Level station ID 1762) and from the Port of Fremantle (Permanent Service for Mean Sea Level station ID 111), obtaining the mean, minimum and maximum monthly sea level from the Bureau of Meteorology, Australia from which an annual mean sea level for each monitoring year (between 1 July and July of each year) and each calendar was calculated. The relationship between normalized salinity of the soil porewater and mean sea level was assessed using regression analysis. Mean annual minimum and maximum sea level was highly correlated with mean annual sea level for any given year, and thus we used the mean annual sea level in our analyses. We tested the difference in soil porewater salinity between live and dead plots monitored in 2016 using a one-way analysis of variance. The relationship between the number of seedlings observed and the soil porewater salinity of the plots in the preceding year, when propagules were developing, was assessed using regression analysis.

The normalized difference vegetation index (NDVI) has been employed in several studies as a proxy to monitor temporal mangrove canopy as it has been shown to highly correlate with canopy closure in mangroves (r = 0.91)^31^. Often used as a ‘greenness’ index, NDVI measures the absorbance of the red band by chlorophyll and the reflection of the near-infrared by the mesophyll leaf structure^32^. As such it has been used to monitor deforestation and degradation^33, 34^ and responses to climatic disturbances, such as cyclones in mangrove forests^35, 36^. NDVI values range from −1 to +1, where any value below zero does not correspond to green vegetation. Dense closed canopy tropical forests tend to produce values closer to 1, whilst sparsely vegetated areas, or open shrub tend to have lower values (0.2–0.3)^35^. Seasonal variations may occur; however, a healthy dense mangrove canopy produces maximum values of approximately 0.7^36^.

1 $${\rm{NDVI}}={\rm{NIR}}-{\rm{Red}}/{\rm{NIR}}+{\rm{Red}}$$

Cloud-free Landsat 5 Thematic Mapper (TM), Landsat 7 and Landsat 8 Operational Land Imager (OLI) imagery (p115r75) was acquired to interrogate field sampling across the entire mangrove extent (Supplementary data Table 1). Atmospheric corrections were performed to convert the pixels to top of atmosphere reflectance. ESRI ArcGIS was used to calculate the Normalized Difference Vegetation Index (NVDI) (Equation 1) (30 m pixel) for the study region. True and false colour scene composites were used to visually determine NDVI minimum and maximum thresholds for mangroves each year. An accuracy assessment from high-resolution aerial imagery for available years (1999, 2003, 2010) was undertaken and Cohen’s kappa was used to measure agreement, k = 0.800 (95%CI, 0.784 to 0.816), p < 0.0005. Euclidean distance to each pixel centroid was also measured from the creek entrance at the northern site. The relationship between mean annual NDVI, sea level and rainfall was assessed using linear regression. We used an F-test to assess whether a linear model including annual rainfall as well as mean annual sea level explained a significantly greater proportion of the variation in annual NDVI compared to a model with only sea level as the explanatory variable. The analysis of the relationship between distance from the mouth of the creek and the coefficient of variation of NDVI and mean NDVI for individual pixels were assessed using linear regression.

## Electronic supplementary material

Supplementary data
