# Supplementary material for: Mangrove dieback during fluctuating sea levels
Source: Sci Rep. 2017 May 10;7:1680. doi: 10.1038/s41598-017-01927-6 (PMC5431776; doi:10.1038/s41598-017-01927-6)
Supplement: Supplementary file 1 — Supplementary data [file 41598_2017_1927_MOESM1_ESM.doc]

Supplementary data

**Mangrove dieback during fluctuating sea levels**

Catherine E. Lovelock^1,*^, Ilka C. Feller^2^, Ruth Reef^3^, Sharyn Hickey^4^, and Marilyn C. Ball^5^

^1^School of Biological Sciences, The University of Queensland, St Lucia, Queensland 4072, Australia.

^2^Smithsonian Environmental Research Center, Edgewater, Maryland 21037, USA.

^3^School of Earth, Atmosphere and Environment, Monash University, Clayton, Victoria 3800, Australia.

^4^ School of Earth and Environment Sciences, Oceans Institute, University of Western Australia, Crawley, Western Australia 6009, Australia.

^5^ Research School of Biology, The Australian National University, Acton, Australian Capital Territory 2601, Australia.

*Correspondence to: [c.lovelock@uq.edu.au](mailto:c.lovelock@uq.edu.au)


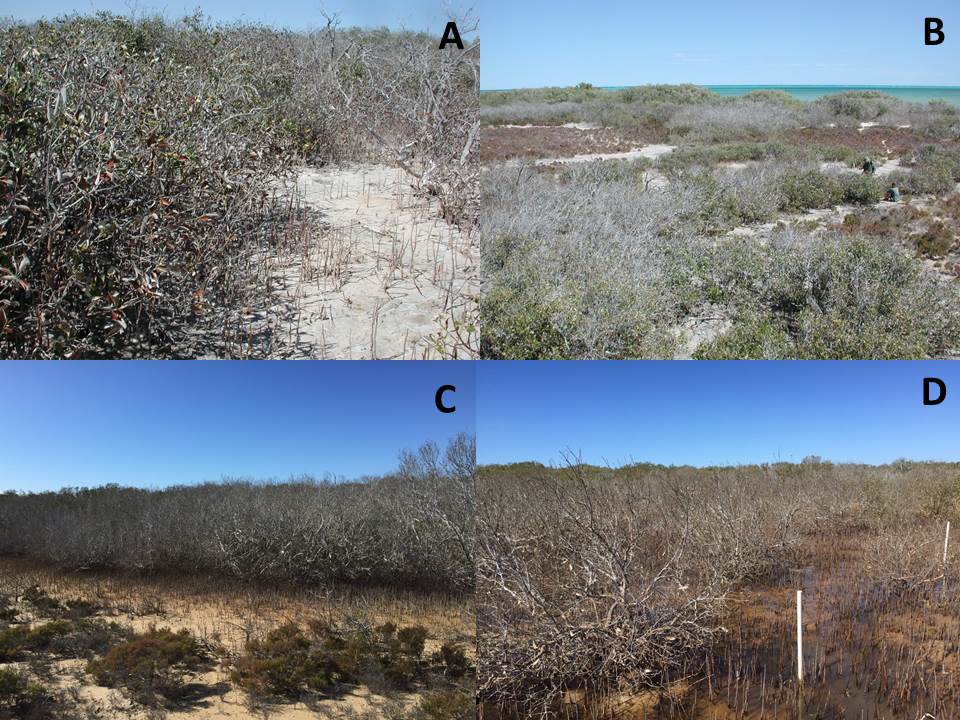


**Supplementary Data Fig. 1** Images of the dieback event in 2002-2003 (A and B) and in 2015-2016 (C and D). In panel B, mangrove is interspersed among red saltmarsh vegetation. The figure standing in the right of the image is 1.9 m tall. Live mangrove canopy is evident on the seaward edge in the background. In Panel D, the white poles mark a permanent plot and are 5 m apart. Live canopy can be seen in the background on the lagoon edge. Images are provided by the authors: A and B by Ilka C. Feller and C and D by Catherine E. Lovelock.


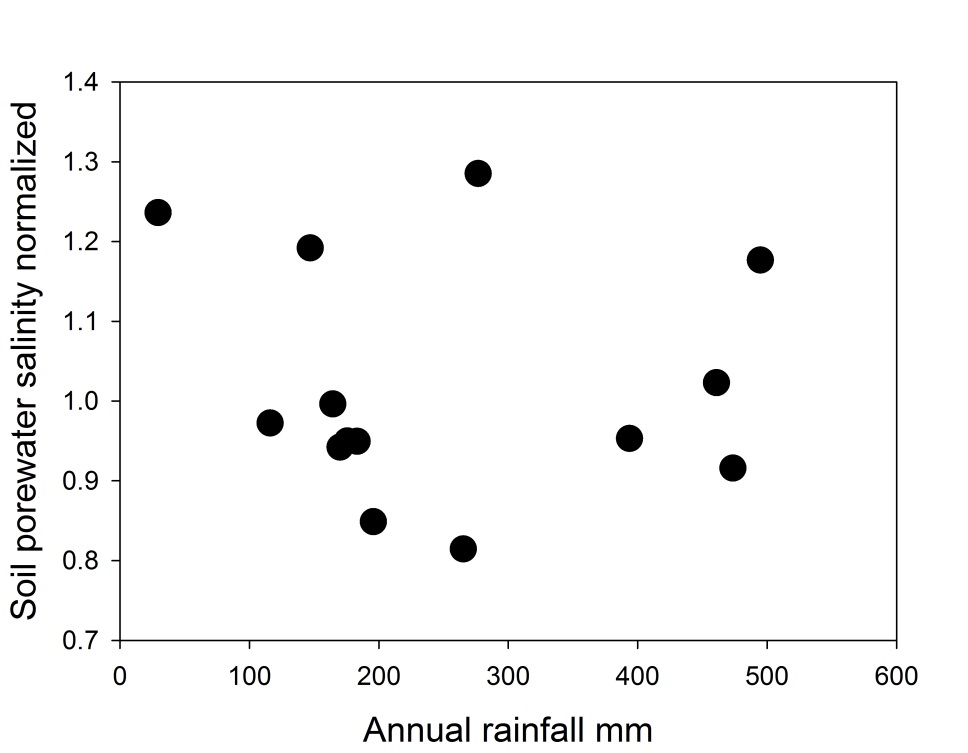


**Supplementary Data Fig. 2** Plot of the normalized soil porewater salinity as a function of annual rainfall. There was no significant relationship (P>0.05).

**
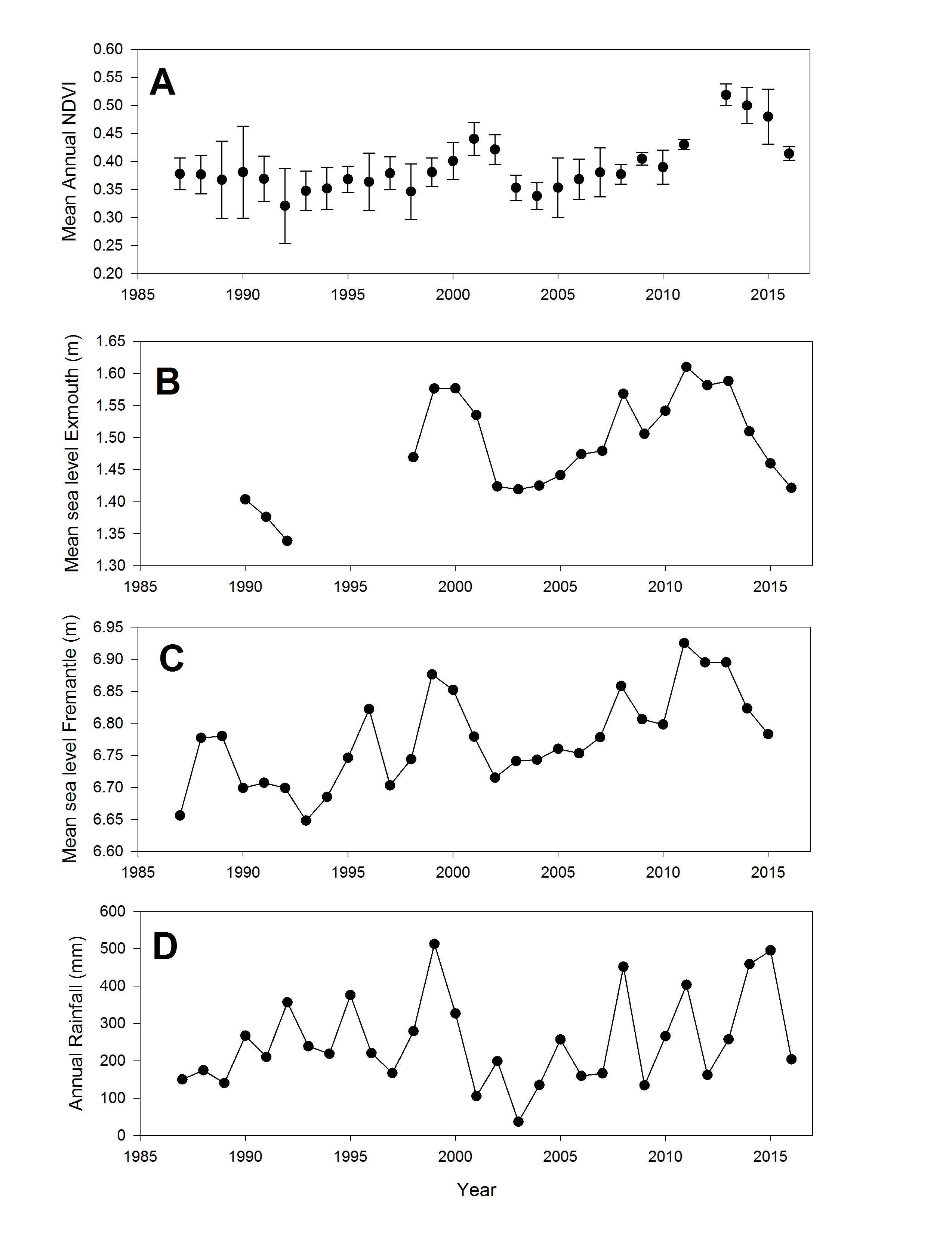
**

**Supplementary Data Fig. 3**.Mean annual NDVI (±standard deviation) (A), annual mean sea level at Exmouth (B), annual mean sea level at Fremantle (C) and annual rainfall at Exmouth (D) from 1987 to 2016.

**Supplementary Data Tables**

**Supplementary Data Table 1.** Landsat scenes used to derive normalized difference vegetation index (NDVI) values for mangrove forests at Mangrove Bay.

| Scene | Month | Year | Landsat |
| --- | --- | --- | --- |
| LT51150751987149ASA00 | 5 | 1987 | Landsat 5 |
| LT51150751987229ASA00 | 8 | 1987 | Landsat 5 |
| LT51150751987261ASA00 | 9 | 1987 | Landsat 5 |
| LT51150751987293ASA00 | 10 | 1987 | Landsat 5 |
| LT51150751987325ASA00 | 11 | 1987 | Landsat 5 |
| LT51150751987341ASA00 | 12 | 1987 | Landsat 5 |
| LT51150751988008ASA00 | 1 | 1988 | Landsat 5 |
| LT51150751988056ASA00 | 2 | 1988 | Landsat 5 |
| LT51150751988072ASA00 | 3 | 1988 | Landsat 5 |
| LT51150751988104ASA00 | 4 | 1988 | Landsat 5 |
| LT51150751988152ASA00 | 5 | 1988 | Landsat 5 |
| LT51150751988168ASA00 | 6 | 1988 | Landsat 5 |
| LT51150751988200ASA00 | 7 | 1988 | Landsat 5 |
| LT51150751988232ASA00 | 8 | 1988 | Landsat 5 |
| LT51150751988264ASA00 | 9 | 1988 | Landsat 5 |
| LT51150751988296ASA00 | 10 | 1988 | Landsat 5 |
| LT51150751988328ASA00 | 11 | 1988 | Landsat 5 |
| LT51150751988360ASA00 | 12 | 1988 | Landsat 5 |
| LT51150751989026ASA00 | 1 | 1989 | Landsat 5 |
| LT51150751989042ASA00 | 2 | 1989 | Landsat 5 |
| LT51150751989074ASA00 | 3 | 1989 | Landsat 5 |
| LT51150751989138ASA00 | 5 | 1989 | Landsat 5 |
| LT51150751989170ASA00 | 6 | 1989 | Landsat 5 |
| LT51150751989186ASA00 | 7 | 1989 | Landsat 5 |
| LT51150751989218ASA01 | 8 | 1989 | Landsat 5 |
| LT51150751989266ASA00 | 9 | 1989 | Landsat 5 |
| LT51150751989282ASA00 | 10 | 1989 | Landsat 5 |
| LT51150751989330ASA00 | 11 | 1989 | Landsat 5 |
| LT51150751989362ASA00 | 12 | 1989 | Landsat 5 |
| LT51150751990109ASA00 | 4 | 1990 | Landsat 5 |
| LT51150751990141ASA00 | 5 | 1990 | Landsat 5 |
| LT51150751990173ASA00 | 6 | 1990 | Landsat 5 |
| LT51150751990205ASA00 | 7 | 1990 | Landsat 5 |
| LT51150751990237ASA00 | 8 | 1990 | Landsat 5 |
| LT51150751990253ASA00 | 9 | 1990 | Landsat 5 |
| LT51150751990285ASA00 | 10 | 1990 | Landsat 5 |
| LT51150751990333ASA00 | 11 | 1990 | Landsat 5 |
| LT51150751990349ASA00 | 12 | 1990 | Landsat 5 |
| LT51150751991048ASA00 | 2 | 1991 | Landsat 5 |
| LT51150751991128ASA00 | 5 | 1991 | Landsat 5 |
| LT51150751991192ASA00 | 7 | 1991 | Landsat 5 |
| LT51150751991224ASA00 | 8 | 1991 | Landsat 5 |
| LT51150751991256ASA00 | 9 | 1991 | Landsat 5 |
| LT51150751991288ASA00 | 10 | 1991 | Landsat 5 |
| LT51150751991320ASA00 | 11 | 1991 | Landsat 5 |
| LT51150751991336ASA00 | 12 | 1991 | Landsat 5 |
| LT51150751992003ASA00 | 1 | 1992 | Landsat 5 |
| LT51150751992035ASA00 | 2 | 1992 | Landsat 5 |
| LT51150751992067ASA00 | 3 | 1992 | Landsat 5 |
| LT51150751992099ASA00 | 4 | 1992 | Landsat 5 |
| LT51150751992131ASA00 | 5 | 1992 | Landsat 5 |
| LT51150751992179ASA00 | 6 | 1992 | Landsat 5 |
| LT51150751992195ASA00 | 7 | 1992 | Landsat 5 |
| LT51150751992227ASA00 | 8 | 1992 | Landsat 5 |
| LT51150751992259ASA00 | 9 | 1992 | Landsat 5 |
| LT51150751992339ASA00 | 12 | 1992 | Landsat 5 |
| LT51150751993005ASA00 | 1 | 1993 | Landsat 5 |
| LT51150751993053ASA00 | 2 | 1993 | Landsat 5 |
| LT51150751993069ASA00 | 3 | 1993 | Landsat 5 |
| LT51150751993117ASA00 | 4 | 1993 | Landsat 5 |
| LT51150751993165ASA00 | 6 | 1993 | Landsat 5 |
| LT51150751993197ASA00 | 7 | 1993 | Landsat 5 |
| LT51150751993229ASA00 | 8 | 1993 | Landsat 5 |
| LT51150751993245ASA00 | 9 | 1993 | Landsat 5 |
| LT51150751993277ASA00 | 10 | 1993 | Landsat 5 |
| LT51150751993325ASA00 | 11 | 1993 | Landsat 5 |
| LT51150751994008ASA00 | 1 | 1994 | Landsat 5 |
| LT51150751994040ASA00 | 2 | 1994 | Landsat 5 |
| LT51150751994072ASA01 | 3 | 1994 | Landsat 5 |
| LT51150751994136ASA00 | 5 | 1994 | Landsat 5 |
| LT51150751994168ASA00 | 6 | 1994 | Landsat 5 |
| LT51150751994184ASA00 | 7 | 1994 | Landsat 5 |
| LT51150751994232ASA00 | 8 | 1994 | Landsat 5 |
| LT51150751994248ASA00 | 9 | 1994 | Landsat 5 |
| LT51150751994296ASA00 | 10 | 1994 | Landsat 5 |
| LT51150751994312ASA00 | 11 | 1994 | Landsat 5 |
| LT51150751994360ASA00 | 12 | 1994 | Landsat 5 |
| LT51150751995011ASA00 | 1 | 1995 | Landsat 5 |
| LT51150751995075ASA00 | 3 | 1995 | Landsat 5 |
| LT51150751995107ASA00 | 4 | 1995 | Landsat 5 |
| LT51150751995139ASA00 | 5 | 1995 | Landsat 5 |
| LT51150751995155ASA00 | 6 | 1995 | Landsat 5 |
| LT51150751995203ASA00 | 7 | 1995 | Landsat 5 |
| LT51150751995219ASA00 | 8 | 1995 | Landsat 5 |
| LT51150751995299ASA00 | 10 | 1995 | Landsat 5 |
| LT51150751995315ASA00 | 11 | 1995 | Landsat 5 |
| LT51150751995363ASA00 | 12 | 1995 | Landsat 5 |
| LT51150751996014ASA00 | 1 | 1996 | Landsat 5 |
| LT51150751996046ASA00 | 2 | 1996 | Landsat 5 |
| LT51150751996078ASA00 | 3 | 1996 | Landsat 5 |
| LT51150751996094ASA00 | 4 | 1996 | Landsat 5 |
| LT51150751996142ASA00 | 5 | 1996 | Landsat 5 |
| LT51150751996174ASA00 | 6 | 1996 | Landsat 5 |
| LT51150751996222ASA00 | 8 | 1996 | Landsat 5 |
| LT51150751996270ASA00 | 9 | 1996 | Landsat 5 |
| LT51150751996286ASA00 | 10 | 1996 | Landsat 5 |
| LT51150751996318ASA00 | 11 | 1996 | Landsat 5 |
| LT51150751996350ASA00 | 12 | 1996 | Landsat 5 |
| LT51150751997016ASA00 | 1 | 1997 | Landsat 5 |
| LT51150751997064ASA00 | 3 | 1997 | Landsat 5 |
| LT51150751997112ASA00 | 4 | 1997 | Landsat 5 |
| LT51150751997144ASA00 | 5 | 1997 | Landsat 5 |
| LT51150751997192ASA00 | 7 | 1997 | Landsat 5 |
| LT51150751997224ASA00 | 8 | 1997 | Landsat 5 |
| LT51150751997304ASA00 | 10 | 1997 | Landsat 5 |
| LT51150751997352ASA00 | 12 | 1997 | Landsat 5 |
| LT51150751998003ASA00 | 1 | 1998 | Landsat 5 |
| LT51150751998035ASA00 | 2 | 1998 | Landsat 5 |
| LT51150751998067ASA00 | 3 | 1998 | Landsat 5 |
| LT51150751998115ASA00 | 4 | 1998 | Landsat 5 |
| LT51150751998179ASA00 | 6 | 1998 | Landsat 5 |
| LT51150751998243ASA00 | 8 | 1998 | Landsat 5 |
| LT51150751998259ASA00 | 9 | 1998 | Landsat 5 |
| LT51150751998291ASA00 | 10 | 1998 | Landsat 5 |
| LT51150751998323ASA00 | 11 | 1998 | Landsat 5 |
| LT51150751998355ASA00 | 12 | 1998 | Landsat 5 |
| LT51150751999054ASA00 | 2 | 1999 | Landsat 5 |
| LT51150751999070ASA00 | 3 | 1999 | Landsat 5 |
| LT51150751999102ASA00 | 4 | 1999 | Landsat 5 |
| LT51150751999166ASA00 | 6 | 1999 | Landsat 5 |
| LT51150751999198ASA00 | 7 | 1999 | Landsat 5 |
| LT51150751999230ASA00 | 8 | 1999 | Landsat 5 |
| LT51150751999262ASA00 | 9 | 1999 | Landsat 5 |
| LT51150751999294ASA00 | 10 | 1999 | Landsat 5 |
| LT51150751999310ASA00 | 11 | 1999 | Landsat 5 |
| LT51150751999358ASA00* | 12 | 1999 | Landsat 5 |
| LE71150752000017SGS00 | 1 | 2000 | Landsat 7 |
| LE71150752000065ASA00 | 3 | 2000 | Landsat 7 |
| LE71150752000097ASA00 | 4 | 2000 | Landsat 7 |
| LE71150752000225ASA00 | 8 | 2000 | Landsat 7 |
| LE71150752000337ASA00 | 12 | 2000 | Landsat 7 |
| LE71150752001131ASA00 | 5 | 2001 | Landsat 7 |
| LE71150752001243ASA00 | 8 | 2001 | Landsat 7 |
| LE71150752001259ASA00 | 9 | 2001 | Landsat 7 |
| LE71150752002006SGS00 | 1 | 2002 | Landsat 7 |
| LE71150752002038ASA00 | 2 | 2002 | Landsat 7 |
| LE71150752002070ASA00 | 3 | 2002 | Landsat 7 |
| LE71150752002102SGS00 | 4 | 2002 | Landsat 7 |
| LE71150752002198ASA00 | 7 | 2002 | Landsat 7 |
| LE71150752002342ASA00 | 12 | 2002 | Landsat 7 |
| LE71150752003057ASN01 | 2 | 2003 | Landsat 7 |
| LE71150752003089ASA00 | 3 | 2003 | Landsat 7 |
| LT51150752003193ASA01 | 7 | 2003 | Landsat 5 |
| LT51150752003225ASA01 | 8 | 2003 | Landsat 5 |
| LT51150752003257ASA01 | 9 | 2003 | Landsat 5 |
| LT51150752003289ASA01 | 10 | 2003 | Landsat 5 |
| LT51150752003305ASA01 | 11 | 2003 | Landsat 5 |
| LT51150752003353ASA01* | 12 | 2003 | Landsat 5 |
| LT51150752004020ASA01 | 1 | 2004 | Landsat 5 |
| LT51150752004036ASA01 | 2 | 2004 | Landsat 5 |
| LT51150752004068ASA01 | 3 | 2004 | Landsat 5 |
| LT51150752004116ASA01 | 4 | 2004 | Landsat 5 |
| LT51150752004132ASA01 | 5 | 2004 | Landsat 5 |
| LT51150752004164XXX01 | 6 | 2004 | Landsat 5 |
| LT51150752004196ASA00 | 7 | 2004 | Landsat 5 |
| LT51150752004228ASA00 | 8 | 2004 | Landsat 5 |
| LT51150752004260ASA00 | 9 | 2004 | Landsat 5 |
| LT51150752004292ASA00 | 10 | 2004 | Landsat 5 |
| LT51150752004324ASA00 | 11 | 2004 | Landsat 5 |
| LT51150752004356ASA00 | 12 | 2004 | Landsat 5 |
| LT51150752005022ASA02 | 1 | 2005 | Landsat 5 |
| LT51150752005054ASA01 | 2 | 2005 | Landsat 5 |
| LT51150752005134ASA01 | 5 | 2005 | Landsat 5 |
| LT51150752005166ASA01 | 6 | 2005 | Landsat 5 |
| LT51150752005198ASA01 | 7 | 2005 | Landsat 5 |
| LT51150752005230ASA01 | 8 | 2005 | Landsat 5 |
| LT51150752005262ASA01 | 9 | 2005 | Landsat 5 |
| LT51150752005278ASA01 | 10 | 2005 | Landsat 5 |
| LT51150752005310ASA01 | 11 | 2005 | Landsat 5 |
| LT51150752006041ASA00 | 2 | 2006 | Landsat 5 |
| LT51150752006073ASA00 | 3 | 2006 | Landsat 5 |
| LT51150752006105ASA00 | 4 | 2006 | Landsat 5 |
| LT51150752006137ASA01 | 5 | 2006 | Landsat 5 |
| LT51150752006169ASA01 | 6 | 2006 | Landsat 5 |
| LT51150752006201ASA00 | 7 | 2006 | Landsat 5 |
| LT51150752006217ASA00 | 8 | 2006 | Landsat 5 |
| LT51150752006265ASA00 | 9 | 2006 | Landsat 5 |
| LT51150752006281ASA00 | 10 | 2006 | Landsat 5 |
| LT51150752006313ASA00 | 11 | 2006 | Landsat 5 |
| LT51150752006345ASA00 | 12 | 2006 | Landsat 5 |
| LT51150752007028ASA00 | 1 | 2007 | Landsat 5 |
| LT51150752007060ASA00 | 3 | 2007 | Landsat 5 |
| LT51150752007108ASA00 | 4 | 2007 | Landsat 5 |
| LT51150752007172ASA00 | 6 | 2007 | Landsat 5 |
| LT51150752007188ASA00 | 7 | 2007 | Landsat 5 |
| LT51150752007220ASA00 | 8 | 2007 | Landsat 5 |
| LT51150752007252ASA00 | 9 | 2007 | Landsat 5 |
| LT51150752008047ASA00 | 2 | 2008 | Landsat 5 |
| LT51150752008287ASA00 | 10 | 2008 | Landsat 5 |
| LT51150752008319ASA00 | 11 | 2008 | Landsat 5 |
| LT51150752009017ASA00 | 1 | 2009 | Landsat 5 |
| LT51150752009081ASA00 | 3 | 2009 | Landsat 5 |
| LT51150752009129ASA00 | 5 | 2009 | Landsat 5 |
| LT51150752009289ASA00 | 10 | 2009 | Landsat 5 |
| LT51150752009321ASA00 | 11 | 2009 | Landsat 5 |
| LT51150752010020ASA00 | 1 | 2010 | Landsat 5 |
| LT51150752010052ASA00 | 2 | 2010 | Landsat 5 |
| LT51150752010132ASA00 | 5 | 2010 | Landsat 5 |
| LT51150752010180ASA00 | 6 | 2010 | Landsat 5 |
| LT51150752010356ASA00* | 12 | 2010 | Landsat 5 |
| LT51150752011087ASA00 | 3 | 2011 | Landsat 5 |
| LT51150752011119ASA00 | 4 | 2011 | Landsat 5 |
| LT51150752011167ASA00 | 6 | 2011 | Landsat 5 |
| LC81150752013108LGN01 | 4 | 2013 | Landsat 8 |
| LC81150752013124LGN01 | 5 | 2013 | Landsat 8 |
| LC81150752013172LGN00 | 6 | 2013 | Landsat 8 |
| LC81150752013188LGN00 | 7 | 2013 | Landsat 8 |
| LC81150752013220LGN00 | 8 | 2013 | Landsat 8 |
| LC81150752013252LGN00 | 9 | 2013 | Landsat 8 |
| LC81150752013284LGN00 | 10 | 2013 | Landsat 8 |
| LC81150752013316LGN00 | 11 | 2013 | Landsat 8 |
| LC81150752013348LGN00 | 12 | 2013 | Landsat 8 |
| LC81150752014031LGN00 | 1 | 2014 | Landsat 8 |
| LC81150752014047LGN00 | 2 | 2014 | Landsat 8 |
| LO81150752014063LGN00 | 3 | 2014 | Landsat 8 |
| LC81150752014095LGN00 | 4 | 2014 | Landsat 8 |
| LC81150752014143LGN00 | 5 | 2014 | Landsat 8 |
| LC81150752014191LGN00 | 7 | 2014 | Landsat 8 |
| LC81150752014223LGN00 | 8 | 2014 | Landsat 8 |
| LC81150752014255LGN00 | 9 | 2014 | Landsat 8 |
| LC81150752014287LGN00 | 10 | 2014 | Landsat 8 |
| LC81150752014319LGN00 | 11 | 2014 | Landsat 8 |
| LC81150752014351LGN00 | 12 | 2014 | Landsat 8 |
| LC81150752015034LGN00 | 2 | 2015 | Landsat 8 |
| LC81150752015114LGN00 | 4 | 2015 | Landsat 8 |
| LC81150752015130LGN00 | 5 | 2015 | Landsat 8 |
| LC81150752015162LGN00 | 6 | 2015 | Landsat 8 |
| LC81150752015210LGN00 | 7 | 2015 | Landsat 8 |
| LC81150752015242LGN00 | 8 | 2015 | Landsat 8 |
| LC81150752015258LGN00 | 9 | 2015 | Landsat 8 |
| LC81150752015274LGN00 | 10 | 2015 | Landsat 8 |
| LC81150752015322LGN00 | 11 | 2015 | Landsat 8 |
| LC81150752015354LGN00 | 12 | 2015 | Landsat 8 |
| LC81150752016037LGN01 | 2 | 2016 | Landsat 8 |
| LC81150752016069LGN00 | 3 | 2016 | Landsat 8 |
| LC81150752016117LGN00 | 4 | 2016 | Landsat 8 |
| LC81150752016133LGN00 | 5 | 2016 | Landsat 8 |

*Scenes used in accuracy assessment

**Supplementary Data Table 2.** Data set presented in Figs. 2 - 4. Annual values are calculated from July each year to match the monitoring campaigns which occurred in the austral winter (July – August).

| Year of census | Mean soil porewater salinity (ppt) –Fertilization experiment | std dev (site 1, *N* =21 trees) | Mean soil porewater salinity (ppt) – permanent plots | std dev (plots, *N* = 12 plots) | Normalized salinity | Total seedling count | Annual mean sea level (m) | Annual rainfall mm |
| --- | --- | --- | --- | --- | --- | --- | --- | --- |
| 2001 | 71.5 | 5.7 |  |  | 0.97 |  | 1.55 | 116.0 |
| 2002 | 62.4 | 4.8 |  |  | 0.85 |  | 1.47 | 195.8 |
| 2003 | 90.9 | 14.5 |  |  | 1.24 |  | 1.41 | 29.4 |
| 2004 | 89.7 | 6.5 |  |  | 1.19 |  | 1.43 | 147.0 |
| 2005 |  |  |  |  |  |  | 1.42 | 233.0 |
| 2006 | 71.6 | 3.8 |  |  | 0.95 | 973 | 1.48 | 175.6 |
| 2007 | 65.9 | 7.8 | 54.7 | 6.7 | 0.94 | 767 | 1.45 | 170.0 |
| 2008 | 73.0 | 6.4 | 54.8 | 8.0 | 0.95 | 307 | 1.55 | 393.8 |
| 2009 |  |  |  |  |  |  | 1.54 | 197.0 |
| 2010 | 71.7 | 5.2 | 55.4 | 8.8 | 0.95 | 418 | 1.45 | 183.2 |
| 2011 |  |  | 54.2 | 4.5 | 0.92 | 225 | 1.65 | 473.6 |
| 2012 |  |  | 58.9 | 7.2 | 1.00 | 271 | 1.59 | 164.4 |
| 2013 |  |  | 48.2 | 7.7 | 0.81 | 275 | 1.58 | 265.2 |
| 2014 |  |  | 60.5 | 5.7 | 1.02 | 1234 | 1.53 | 461.0 |
| 2015 |  |  | 69.6 | 11.5 | 1.18 | 49 | 1.49 | 494.8 |
| 2016 |  |  | 76.0 | 13.3 | 1.28 | 5 | 1.42 | 276.8 |

**Supplementary Data Table 3.** F-test to assess whether variation in mean annual Normalized Difference Vegetation Index (NDVI) at Mangrove Bay, Western Australia from 1987-2016 could better explained by a model that included both mean annual sea level and annual rainfall, compared to a simpler model that only included mean sea level. The F ratio was smaller than F_critical_, thus the more complex model did not explain significantly more of the variation in mean annual NDVI than the simpler model.

| Model variables | Mean sum of squares | Degrees of Freedom | F |
| --- | --- | --- | --- |
| Sea level + Rainfall | 0.043224 | 25 |  |
|  |  |  |  |
| Sea level | 0.043724 | 26 | 0.289 |
